# A Seven-Long Non-coding RNA Signature Improves Prognosis Prediction of Lung Adenocarcinoma: An Integrated Competing Endogenous RNA Network Analysis

**DOI:** 10.3389/fgene.2020.625977

**Published:** 2021-01-28

**Authors:** Rang Li, Kedong Han, Dehua Xu, Xiaolin Chen, Shujin Lan, Yuanjun Liao, Shengnan Sun, Shaoqi Rao

**Affiliations:** ^1^Institute of Medical Systems Biology, School of Public Health, Guangdong Medical University, Dongguan, China; ^2^Department of Cardiology, Maoming People’s Hospital, Maoming, China

**Keywords:** lung adenocarcinoma, prognosis, lncRNA, molecular signature for survival, ceRNA network

## Abstract

Early and precise prediction is an important way to reduce the poor prognosis of lung adenocarcinoma (LUAD) patients. Nevertheless, the widely used tumor, node, and metastasis (TNM) staging system based on anatomical information only often could not achieve adequate performance on foreseeing the prognosis of LUAD patients. This study thus aimed to examine whether the long non-coding RNAs (lncRNAs), known highly involved in the tumorigenesis of LUAD through the competing endogenous RNAs (ceRNAs) mechanism, could provide additional information to improve prognosis prediction of LUAD patients. To prove the hypothesis, a dataset consisting of both RNA sequencing data and clinical pathological data, obtained from The Cancer Genome Atlas (TCGA) database, was analyzed. Then, differentially expressed RNAs (DElncRNAs, DEmiRNAs, and DEmRNAs) were identified and a lncRNA–miRNA–mRNA ceRNA network was constructed based on those differentially expressed RNAs. Functional enrichment analysis revealed that this ceRNA network was highly enriched in some cancer-associated signaling pathways. Next, lasso-Cox model was run 1,000 times to recognize the potential survival-related combinations of the candidate lncRNAs in the ceRNA network, followed by the “best subset selection” to further optimize these lncRNA-based combinations, and a seven-lncRNA prognostic signature with the best performance was determined. Based on the median risk score, LUAD patients could be well distinguished into high-/low-risk subgroups. The Kaplan–Meier survival curve showed that LUAD patients in the high-risk group had significantly shorter overall survival than those in the low-risk group (log-rank test *P* = 4.52 × 10^–9^). The ROC curve indicated that the clinical genomic model including both the TNM staging system and the signature had a superior performance in predicting the patients’ overall survival compared to the clinical model with the TNM staging system only. Further stratification analysis suggested that the signature could work well in the different strata of the stage, gender, or age, rendering it to be a wide application. Finally, a ceRNA subnetwork related to the signature was extracted, demonstrating its high involvement in the tumorigenesis mechanism of LUAD. In conclusion, the present study established a lncRNA-based molecular signature, which can significantly improve prognosis prediction for LUAD patients.

## Introduction

Lung adenocarcinoma (LUAD), a major type of non-small cell lung cancer (NSCLC), has a low survival rate and an increasing incidence (Matsuda and Machii, 2015; Denisenko et al., 2018). The etiology of LUAD is multifactorial, involving a large number of environmental factors and internal factors (Rajer et al., 2014). So far, because of the lack of specific symptoms and signs, LUAD coupling with complex and diverse clinical manifestations is easily missed and misdiagnosed (Del et al., 2017). Hence, when most LUAD patients are diagnosed, they are already in an advanced stage. Although many kinds of treatments, including surgical resection, chemotherapy, radiotherapy, and chemo-radiotherapy, were applied to improve patient’s survival rate, the overall 5-year survival rate is still extremely bleak (Wakeam et al., 2017). Therefore, early detection and diagnosis is vital to improve LUAD patient’s poor prognosis.

The TNM staging system is currently the most common tumor prognosis predictor and a powerful tool for guiding adjuvant therapy at present (Mittendorf et al., 2015; Pontius et al., 2017). According to the invasion extent of the primary tumor stage (T stage), regional lymph node metastasis stage (N stage), and distant metastasis stage (M stage), the total pathologic stage of the malignant tumor (the TNM stage) could be determined (Hutter, 1991). In general, the higher the TNM stage, the higher the degree of the malignant tumor is. However, the TNM staging system, which is limited to the anatomical extent rather than the biological behavior of the disease, has obvious limitations compared with the multifactorial prognostic index (Fouad et al., 2017; Ball, 2019). Given the shortcomings of the TNM staging system in LUAD patient’s prognosis prediction, it is highly demanding to develop a molecular diagnostic and predictive biomarker.

Long non-coding RNAs (lncRNAs) are defined as any ncRNA that is 200 nucleotides to 100 kb in length (Yin et al., 2018). Many studies reported that lncRNA plays an important role in the pathogenesis of cancer and has significant clinical value in prognosis and diagnosis (Knoll et al., 2015; Evans et al., 2016; Huang et al., 2017; Tripathi et al., 2018). It was also demonstrated that lncRNA can act as a “sponge” to regulate the targeted gene expression by competitively binding with miRNA (Zhang et al., 2016). This novel model of gene regulation is a part of the competing endogenous RNA (ceRNA) hypothesis, which was first proposed in 2011 (Salmena et al., 2011). ceRNAs (including lncRNA, circRNA, and mRNA) competitively bind with microRNA via sharing microRNA response elements (MREs) to weaken the inhibition effect for the target gene. The regulatory relations among lncRNAs, miRNA, and mRNA form a complex ceRNA network, and the abnormal expression of lncRNA would destroy the balance of the ceRNA network to lead to the initiation and progression of cancer (Karreth and Pandolfi, 2013).

Owing to the heterogeneity and polygenic mutation in lung cancer, a single genomic mutation is difficult to explain the various phenotypes and the variable risks of complex disease (Andreassen et al., 2014). Compared with a single gene and single factor, a biomolecular network(s) including multiple disease-related factors, which perform their dysfunctions through physical and biochemical interactions in a network (Zhao and Liu, 2019), represents various molecular relationships underlying complex diseases and depicts a clear global picture of interactions among disease-related factors (Jinawath et al., 2016). As a biomolecular network, the ceRNA network, describing post-transcriptional interactions between ceRNAs and miRNAs, had great value in prognosis, diagnosis, and therapy of cancers (Lin et al., 2018; Zhang Y. et al., 2018; Eissa et al., 2019). In recent years, there are several successful attempts that use the ceRNA networks to identify prognostic signatures for different cancers (Hu et al., 2019; Wang et al., 2019b).

The aim of the present study was to establish a multi-lncRNA prognosis predictor. To this end, a LUAD-related lncRNA–miRNA–mRNA ceRNA network was constructed based on integrated transcriptome data from The Cancer Genome Atlas (TCGA) database. Then, by using lasso-Cox regression model, a seven-lncRNA prognostic signature was identified from the LUAD-related ceRNA network. Survival analysis and the receiver operating characteristic (ROC) curve suggested the seven-lncRNA prognostic signature is a robust and independent prognostic factor. Most importantly, our study demonstrated that the seven-lncRNA prognostic signature effectively enhanced the prognosis prediction performance over the conventional TNM staging system.

## Materials and Methods

### Data Retrieval and Processing

RNA and miRNA sequencing raw count data and corresponding clinical data of LUAD patients were obtained from TCGA database by using the GDC Data Transfer Tool. Then, individual sample expression files were merged into an expression matrix using the Perl language for further processing. To eliminate the adverse effect of low abundance, RNAs with an average value of less than 1 were excluded in further analysis. Finally, the trimmed mean of the *M*-values (TMM) method was used to normalize RNA sequencing data (Smid et al., 2018).

### Identifying Differentially Expressed RNAs

Differentially expressed RNAs (DE-lncRNAs, DE-miRNAs, and DE-mRNAs) were identified by comparing the expression values between LUAD samples and adjacent normal tissue samples based on the edgeR package of R platform (Robinson et al., 2010). The cutoff criterion was set at | logFC| > 1 and FDR < 0.05 for the screening of DE-lncRNAs, DE-miRNAs, and DE-mRNAs. Volcano plot was used to display the differentially expressed RNAs.

### Constructing the ceRNA Network

Regulatory relationships among DE-RNAs were identified by mining knowledge of several public databases. miRcode database (Jeggari et al., 2012) was used to predict the regulatory relationships between lncRNAs and miRNAs, while miRDB, miRTarBase, and TargetScan databases (Zheng et al., 2019) were used to define the regulatory relationship between miRNAs and mRNAs. According to the ceRNA theory, there should be a negative regulatory relationship between ceRNAs and miRNAs (Taulli et al., 2013). Therefore, the Pearson correlation coefficient between lncRNAs/mRNAs and miRNAs was calculated to identify negatively correlated RNA–RNA regulatory pairs (*P* < 0.05). In short, the regulatory relationships between lncRNAs/mRNAs and miRNAs were determined by three facts: (1) having a biological basis, supported by knowledge bases; (2) being a negative relationship, which agrees with the competing endogenous RNA theory; and (3) achieving the significance level in the Pearson correlation analysis based on their expression data. Finally, based on the shared miRNAs among these regulatory pairs, the lncRNA–miRNA–mRNA ceRNA network was built by connecting negative lncRNA–miRNA and miRNA–mRNA regulatory pairs. Cytoscape v3.7.1 was used for network visualization (Shannon et al., 2003).

To reveal the biological function(s) that ceRNA regulatory network involved, the Kyoto Encyclopedia of Genes and Genomes (KEGG)-based enrichment analysis was conducted to assess the ceRNA regulatory network using clusterProfiler package in R (Yu et al., 2012). The enriched KEGG pathway(s) with a FDR less than 0.05 was considered as statistically significant.

### Defining the Prognostic Signature

In order to identify the lncRNAs and optimal subset(s) related to the overall survival of LUAD patients, lasso-Cox model (R package glmnet) was run 1,000 times to recognize the potential survival-related combinations of the candidate lncRNAs in the ceRNA network, followed by the “best subset selection” (the area under the ROC curve, AUC > 0.70 with the minimal set size) to further optimize these lncRNA-based combinations (i.e., the survival-related signatures). Finally, in order to evaluate the joint effect of the best signature, a risk score was calculated based on a linear combination of the expression levels of the included lncRNAs weighted by their regression coefficients derived from the multivariate Cox regression analysis.

The risk score formula was defined as following:

$$R\,i\,s\,k\,s\,c\,o\,r\,e=\sum_{i=1}^{n}\exp_{i}\beta_{i}$$

Here, *n* represents the number of lncRNAs in the model, exp*_*i*_* represents the expression level of lncRNA *i*, and β_*i*_ is the regression coefficient of lncRNA *i* in multivariate Cox regression model.

### Assessing the Prognosis Value of the Newly Identified Signature

First, in order to evaluate its potential to classify the LUAD patients, according to the median risk score of the signature, LUAD patients were divided into low- and high-risk groups. The Kaplan–Meier method was used to display the difference in survival time between low-risk and high-risk LUAD groups. The statistical significance of the difference between the survival profiles of the two groups was determined by using the log-rank test. ROC curve was used to estimate its sensitivity and specificity. Second, to assess whether combining the lncRNA-based signature with the TNM stages could improve the prognosis prediction for LUAD, a clinical genomic model with the TNM stages and the lncRNA-based signature combined was constructed, and AUC was compared to the model with the TNM stages only. Third, in order to explore its applicability, a stratification analysis by the TNM stages, gender, or age was performed. Finally, to explore its biological role(s), a core ceRNA network was constructed and its functional involvements were identified by a KEGG-based enrichment analysis.

**FIGURE 1:**
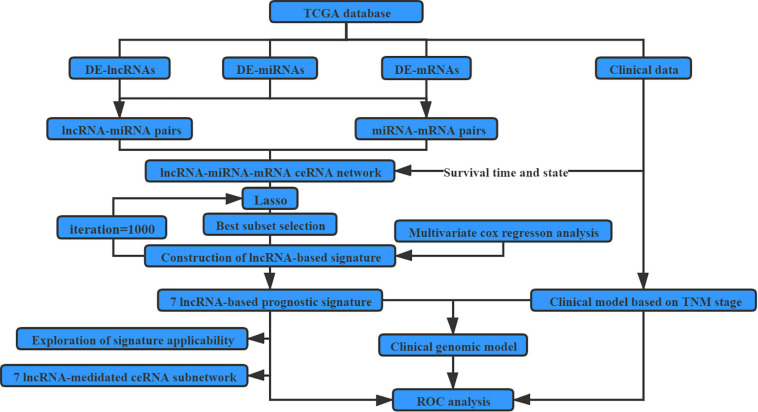
Flow chart for identifying and assessing the survival-related LncRNA-based signature.

The detailed workflows of the proposed strategies for identifying and assessing the survival-related lncRNA-based signature are illustrated in Figure 1.

## Results

### Aberrantly Expressed lncRNAs, miRNAs, and mRNAs

**FIGURE 2:**
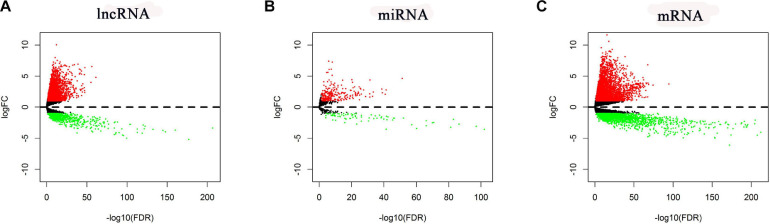
Volcano plots of the DE-lncRNAs, DE-miRNAs, and DE-mRNAs. **(A)** DE-lncRNAs. **(B)** DE-miRNAs. **(C)** DE-mRNAs.

In total, RNA-seq data for 535 LUAD samples and 59 adjacent normal samples, miRNA-seq data for 519 LUAD samples and 48 adjacent normal samples, and the corresponding clinical data for 504 LUAD patients were obtained. According to the cutoff criteria (| logFC| > 1 and FDR < 0.05), 5,537 mRNAs (3,721 upregulated and 1,816 downregulated), 352 miRNAs (273 upregulated and 79 downregulated), and 3,939 lncRNAs (3,202 upregulated and 737 downregulated) were found differentially expressed (named DE-lncRNAs, DE-miRNAs, and DE-mRNAs, respectively). Their volcano plots are shown in Figure 2.

### The ceRNA Network for LUAD

**FIGURE 3:**
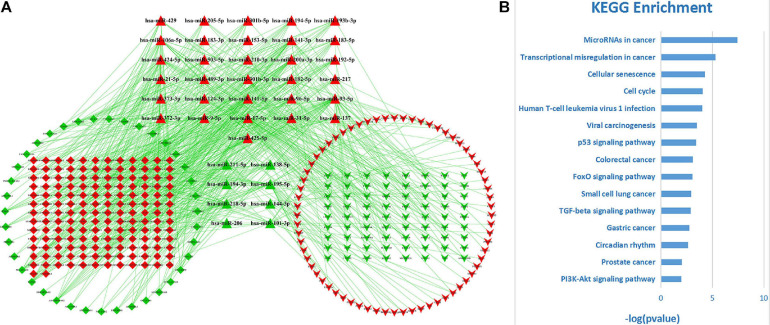
The ceRNA network for LUAD and its functional involvements. **(A)** The topological structure of the ceRNA network for LUAD. Red nodes represent upregulation DE-RNAs and green ones downregulation DE-RNAs. Diamond, triangle, and inverse-triangle represent lncRNA, miRNA, and mRNA, respectively. **(B)** The top 15 KEGG pathways (*P-*value < 0.05) that the network was involved.

Among these DE-RNAs, 475 lncRNA–miRNA pairs between 197 lncRNAs and 39 miRNAs and 198 miRNA–mRNA pairs between 39 miRNAs and 140 mRNAs were found showing a significant negative correlation (*P* < 0.05), after excluding positively correlated pairs. Based on 39 shared miRNAs among these regulatory pairs, the lncRNA–miRNA–mRNA (ceRNA) network was established (Figure 3A). The top 15 KEGG pathways (*P*-value < 0.05) that the network was involved are shown in Figure 3B, indicating that the ceRNA network for LUAD was closely related to some cancer-associated pathways, such as microRNAs in cancer, transcriptional misregulation in cancer, cellular senescence, cell cycle, p53 signaling pathway, small cell lung cancer, and so on.

### The lncRNA-Based Prognostic Signature for LUAD

**FIGURE 4:**
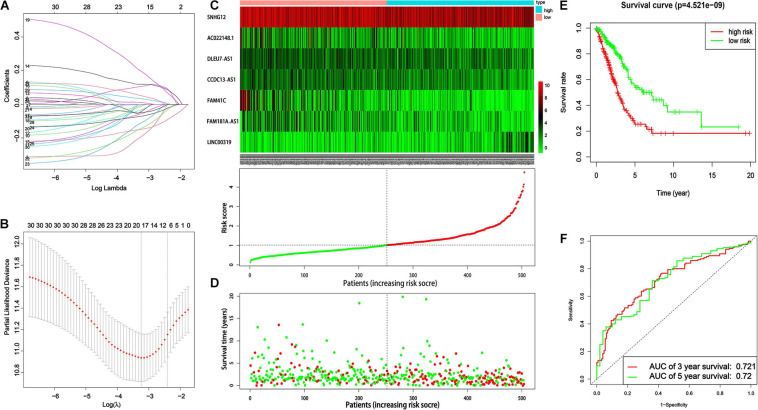
The lncRNA-based prognostic signature for LUAD. **(A)** The convergence of the lasso-based variable selection with the log of the penalty parameter lambda (*λ*). **(B)** The changes of model fitting statistic, partial likelihood deviance, and its range, obtained from 10-fold cross-validation. **(C)** The expression patterns (heat maps) of the seven lncRNAs for patients of two groups (low-risk group, type low and high-risk group, type high). **(D)** The dot plot of survival time for patients sorted by the lncRNA-based risk score. Red dots indicate death events while green ones survivals. **(E)** Kaplan–Meier curves for patients of two categories (low-risk groups versus high-risk groups) defined by the lncRNA-based prognostic signature. **(F)** ROC analysis of the lncRNA-based prognostic signature to estimate AUC values of survival over two different periods.

Among 1,000 lncRNA sets, constructed by using lasso-Cox regression analysis of 197 lncRNAs included in the ceRNA network for LUAD, five had AUC > 0.7. The optimal set with minimal size was selected as the lncRNA-based prognostic signature for LUAD, which was consisted of seven lncRNAs (*SNHG*12, *DLEU*7*_AS1*, *FAM*41*C*, *FAM*181*A_AS*1, *AC*022148.1, *CCDC*13*_AS*1, and *LINC*00319). Figures 4A,B shows the convergence of the lasso-based variable selection (or called feature shrinkage) with the log of the penalty parameter lambda (*λ*) as well as the changes of model fitting statistics (partial likelihood deviance).

Then, a predictive model for the lncRNA set was constructed according to their lncRNA expression values and their corresponding coefficients derived from the multivariate Cox regression analysis. The risk score based on the lncRNA set was defined as following:

$$\begin{array}{cc} \mathrm{Riskscore} & =\left(-0.2196\times S\,N\,H\,G\,12\right)\\ & +\left(-0.14458\times D\,L\,E\,U\,7\,\_\,A\,S\,1\right)\\ & +\left(-0.12641\times F\,A\,M\,41\,C\right)\\ & +\left(-0.08658\times F\,A\,M\,181\,A\,\_\,A\,S\,1\right)\\ & +\left(-0.11915\times A\,C\,022148.1\right)\\ & +\left(-0.15204\times C\,C\,D\,C\,13\,\_\,A\,S\,1\right)\\ & +\left(0.12557\times L\,I\,N\,C\,00319\right) \end{array}$$

Based on the median value of the risk score, 504 patients were subdivided into high-risk and low-risk groups. The expression patterns (heat maps) for patients of two groups (low-risk group and high-risk group) are shown in Figure 4C, while their risk score distribution, as well as their survival, data are shown in Figure 4D, which clearly indicated that with the increase of the risk score, LUAD patients tended to have a shorter survival time. Kaplan–Meier survival analysis showed that the patients in the low-risk group had a better prognosis than those with high-risk scores (*P* = 4.52e^–9^) (Figure 4E). ROC analysis estimated that the AUC values of the lncRNA-based prognostic signature for survival were 0.721 for the third year and 0.72 for the fifth year, respectively (Figure 4F).

### Comparison Between the Clinical Genomic Model With Both the TNM Stages and the lncRNA Signature Included and the Clinical Model With the TNM Stages Only

**FIGURE 5:**
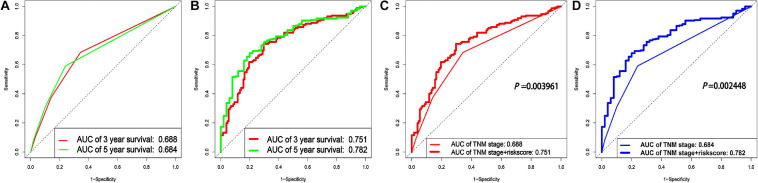
A comparison between the clinical genomic model with both the TNM stages and the lncRNA signature and with the clinical model with the TNM stages only. **(A)** The ROCs of two periods of survival for the conventional model with the TNM stages only. **(B)** The ROCs of two periods of survival for the clinical genomic model with both the TNM stages and the lncRNA-based signature included. **(C,D)** Comparison of the two models for prognosis prediction over the two periods (3 and 5 years), respectively.

In order to verify whether combining the lncRNA-based signature with the TNM stages could improve the prognosis prediction for LUAD, a clinical genomic model with the TNM stages and the lncRNA-based signature combined was constructed, and its area under the ROC curve (AUC) was compared to the model with the TNM stages only. As shown in Figure 5A, the AUC values for the model with the TNM stages only were 0.688 and 0.684 for 3- and 5-year survival, respectively, which were markedly lower than the estimates for the clinical genomic model with both the TNM stages and the lncRNA-based signature (0.751 and 0.782 for the two periods, respectively) (Figure 5B). A comparison of the two models for prognosis prediction over the two periods separately all demonstrated that the clinical genomic model performed significantly better than the conventional model with the TNM stage (all *P* < 0.01) (Figures 5C,D). These results suggest that lncRNAs could provide additional information on the prognosis prediction for LUAD, and more importantly, this fact may render an earlier prognosis prediction for LUAD to be practical.

### Applicability of the lncRNA-Based Prognostic Signature

**FIGURE 6:**
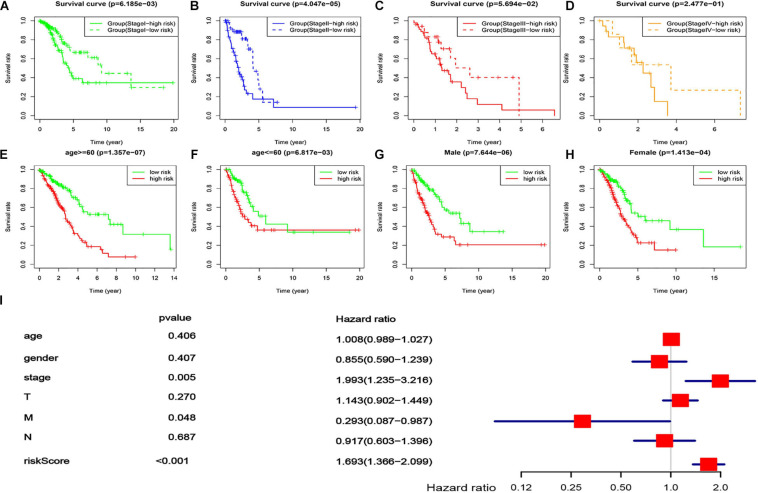
Applicability of the lncRNA-based prognostic signature. **(A–D)** survival curves of the LUAD patients in different risk groups, stratified by four tumor stages [stage I **(A)**, stage II **(B)**, stage III **(C)**, and stage IV **(D)**]. **(E,F)** Survival curves of the LUAD patients in different risk groups, stratified by two ages [age ≤60 **(E)** and age <60 **(F)**]. **(G,H)** Survival curves of the LUAD patients in different risk groups, stratified by gender [male **(G)** and female **(H)**]. **(I)** Multivariate Cox regression with the lncRNA-based signature (risk score) and all available demographic and clinical factors included.

In order to explore its applicability, a stratification analysis by the TNM stages (overall rating, divided into four ranks), gender, or age was performed. The results indicated that the lncRNA-based prognostic signature could work well in most strata of the TNM stages, gender, or age (Figures 6A–C,E–H), i.e., having a good capacity in separating the LUAD patients into high-risk and low-risk groups. For the TNM stages, stage IV was the only exception where no statistical significance was found between the low- and high-risk groups defined by the lncRNA-based signature (Figure 6D), which might be due to the small sample size (*n* = 26). These results suggested that the lncRNA-based prognostic signature was largely independent of tumor stage, gender, and age, which was agreed well with the multivariate Cox regression analysis where the lncRNA-based prognostic signature remained to be the most significant factor (*P* < 0.001) even after adjusting for several demographic and clinical factors (Figure 6I).

### The ceRNA Subnetwork Related to the lncRNA-Based Signature

**FIGURE 7:**
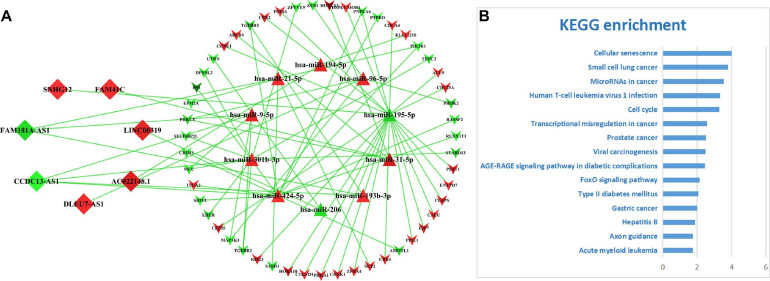
The ceRNA subnetwork mediated by the lncRNA-based signature. **(A)** The ceRNA subnetwork depicting the relationships between seven lncRNAs and their counterparts. Diamonds represent lncRNAs, triangles miRNAs, and inverse-triangles mRNAs. The red color depicts upregulation and green downregulation. **(B)** The top 15 significantly enriched KEGG pathways.

On basis of the seven lncRNAs contained in the prognostic signature for LUAD, a core network (Figure 7A) was extracted from the primary ceRNA network. KEGG-based functional enrichment analysis demonstrated that this lncRNA-mediated ceRNA subnetwork was highly involved in several cancer-associated signaling pathways (Figure 7B), which implicated that the seven lncRNAs play vital roles in the tumorigenesis mechanism of LUAD via regulating related gene expressions by competitively sponging several miRNAs.

## Discussion

As the most common malignancy, LUAD had an unfavorable 5-year survival rate at an advanced stage. Early detection and diagnosis was an important way to improve the LUAD patient’s prognosis. Although it had been confirmed as an effective prognosis predictor for LUAD patients (Carter et al., 2018), the TNM staging system, which was founded on the anatomical information only, could not perfectly perform prognosis prediction. An increasing amount of evidence demonstrated that genetic disorders and alterations were of significance in tumorigenesis and the progression of LUAD, suggesting that molecular markers had great value in the prediction of overall survival of LUAD patients (Trimarchi et al., 2014; Timmer et al., 2016).

With the development of high-throughput sequencing technology, the roles of long non-coding RNAs (lncRNAs) in human cancers had received more and more attention. Previous studies demonstrated that lncRNAs play an important role in tumor proliferation, migration, and invasion (Zhang G. et al., 2018) and have potential value in applications to early prognosis and diagnosis for cancers (Takahashi et al., 2014). Zhai et al. (2017) demonstrated that the lower expression of lncRNA-*SARCC* influenced downstream genes such as *K-RAS*, *MMP-13*, *AKT*, and *P-ERK* expression by suppressing *miR-143-3p* expression, which could enhance RCC cell invasion, migration, and proliferation. Wu et al. (2017) first reported that lncRNA *CASC9*, as an oncogene, promoted ESCC cell growth by negatively regulating *PDCD4* expression via recruiting *EZH2*, which could be a potential diagnosis and prognosis biomarker for ESCC. All these facts highlight the importance of the large number of lncRNAs to be the molecular biomarkers for early prognosis prediction or early diagnosis of cancers.

Compared with the coding RNAs, it was more complex to study the functional meanings of lncRNAs. The ceRNA hypothesis provided a new solution for achieving better functional studies of lncRNAs. In the ceRNA theory, lncRNAs regulate the expression of the targeted genes by competitively absorbing miRNAs at the post-transcriptional level, forming a huge ceRNA regulatory network (Calloni and Bonatto, 2019). In most scenarios, “communications” between ceRNAs and miRNAs were in dynamic balance (Cai and Wan, 2018). However, an abnormal expression of lncRNA destroyed the balance of the ceRNA network, which was closely related to tumorigenesis (Shao et al., 2015). In this study, a total of 3,939 DE-lncRNAs, 352 DE-miRNA, and 5,537 DE-mRNAs were identified. According to the ceRNA theory, the negatively correlated RNA–RNA regulatory pairs were built among DE-lncRNAs, DE-miRNA, and DE-mRNA. Subsequently, a lncRNA–miRNA–mRNA ceRNA network including 197 DE-lncRNAs, 39 DE-miRNAs, and 140 DE-mRNAs was constructed by connecting the negatively correlated RNA–RNA regulatory pairs. Further functional enrichment analysis showed that the ceRNA network was mainly involved in some cancer-related pathways including “microRNAs in cancer,” “transcriptional misregulation in cancer,” “cell cycle,” “p53 signaling pathway,” “colorectal cancer,” “small cell lung cancer,” etc., which was not surprising at all to us because more and more evidence indicated that at the molecular levels various cancers were interconnected. In brief, the ceRNA regulatory network including complex molecular regulatory relationships not only had potential value to mine prognosis-related biomarkers (Bai et al., 2019) but also provided a new avenue to broaden our knowledge on massive lncRNAs and their functional involvements in the pathogenic mechanisms for cancers like LUAD.

Most of the preceding studies focused on single lncRNAs related to lung cancer. Nie et al. (2016) found that *LncRNA-UCA1* upregulated *ERBB4* by sponging *miR-193a-3p* to exert oncogenic functions. Guan et al. (2019) demonstrated that *LINC00673-v4* enhanced cancer cell invasion, migration, and metastasis by overactivating WNT/β-catenin signaling and could be a candidate for the therapeutic target of LUAD patients. Nevertheless, as a complex disease, LUAD was thought to be a series of biological cascades resulting from the perturbations of intracellular and intercellular elements (Fiscon et al., 2018), and it was impossible to have a global picture about the sophisticated pathogenic mechanism of LUAD by studying only a single biomarker. Therefore, compared with a single biomarker, a prognostic signature integrating multiple biomarkers could achieve more power in prognosis prediction for LUAD (Kratz et al., 2019). This study demonstrated that the newly identified lncRNA-based signature with seven lncRNAs could provide >9% improvement in prognosis prediction over various periods for LUAD and was deemed to a robust complement for the conventional TNM staging system.

Among the seven lncRNAs containing the prognostic signature, four (*AC022148.1*, *DLEU7_AS1*, *LINC00319*, and *SNHG12*) were found to be involved in tumorigenesis, migration, and metastasis in cancers. Qi et al. (2019) demonstrated that a seven-lncRNA prognostic model including *AC022148.1* was a robust indicator to assess the prognosis risk of lung squamous cell carcinoma patients. Liu et al. (2018) found that *DLEU7_AS1*, as an adverse prognosis factor, was closely associated with colorectal cancer (CRC) staging, lymph node metastasis, and distant metastasis and may regulate the Wnt/β-catenin pathway to promote the occurrence and development of CRC. Zhou et al. (2017) demonstrated that *LINC00319* strengthened proliferation and invasion of lung cancer cells by downregulating the expression of miR-32 and upregulating the expression levels of miR-32 target genes. Wang et al. (2019a) revealed that knockdown of *SNHG12* inhibited migration and invasion of NSCLC cells via the Slug/zinc finger E-box-binding homeobox 2 EMT signaling pathway by upregulating the expression of miR-218 and could be a potential prognostic marker and therapeutic target for NSCLC. Up to date, there is dearth of information about the roles of the remaining three lncRNAs in tumors, waiting for further studies to clarify.

Compared with several previous studies, our approach has the following improvements. First, for constructing the ceRNA network, we used only negatively correlated lncRNAs/mRNAs–miRNAs regulatory pairs, which fits well the definition of the ceRNA network compared to the previous method without this distinction (Li et al., 2018; Fan et al., 2020; Wu et al., 2020). Second, we applied 1,000 rounds of lasso-Cox regression model fittings to identify the optimal lncRNA-based set, which is deemed to be more robust than the conventional Cox model utilized in a previous study (Zheng et al., 2017). Third, consequently, our study achieved better performance on predicting the survival of the LUAD patients by using a seven-lncRNA-based signature (AUC = 0.72 and 0.721 for 3- and 5-year survival, respectively) than a previous study (Yao et al., 2020), who identified an eight-lncRNA signature for LUAD (AUC = 0.702 and 0.671 for 3- and 5-year survival). Up to date, only the clinical genomic model built by the present study has achieved adequate improvement over the conventional TNM staging system (*P* = 0.003961 and 9.16% for the third year; *P* = 0.002448 and 14.33% for the fifth year), compared to a previous similar study (Zheng et al., 2017), who reported a non-significant improvement (*P* > 0.05 and 4.24% for the fifth year).

In conclusion, we applied an integrated ceRNA network analysis to identify a lncRNA-based signature for predicting the prognosis of LUAD patients. The established molecular signature with seven lncRNAs, derived from the ceRNA network, was demonstrated to be a robust and independent factor for the survival prediction of LUAD patients and, hence, could be an important complement for the conventional TNM staging system.

## Data Availability Statement

The datasets presented in this study can be found in online repositories. The names of the repository/repositories and accession number(s) can be found in the article/supplementary material.

## Author Contributions

SR, RL, and KH conceived and designed the study. RL, KH, SR, YL, SS, and SL performed data analysis. RL, XC, and DX contributed in the software and programming. RL, SR, KH, XC, and DX wrote, reviewed, and edited the manuscript. All authors contributed to the article and approved the submitted version.

## Conflict of Interest

The authors declare that the research was conducted in the absence of any commercial or financial relationships that could be construed as a potential conflict of interest.
